# Water-insensitive down-shifting nanoparticles for sensitive biosensing

**DOI:** 10.1038/s41377-025-01976-x

**Published:** 2025-09-05

**Authors:** Jiang Ming, Sikun Hu, Fan Zhang

**Affiliations:** Department of Chemistry, State Key Laboratory of Molecular Engineering of Polymers, Laboratory of Advanced Materials, Shanghai Key Laboratory of Molecular Catalysis and Innovative Materials and iChem, Fudan University, Shanghai, 200433 China

**Keywords:** Nanoparticles, Imaging and sensing

## Abstract

Conventional optical probes suffer from signal degradation in aqueous media, hindering sensitive biodetection. To overcome this, newly developed water-insensitive down-shifting nanoparticles (WINPs) possess superior photophysical properties in the NIR-I window, including high quantum yield and negligible thermal effects, permitting stable, high-contrast signal generation under low excitation power. This advantage facilitated a low-power lateral flow assay capable of highly sensitive avian influenza virus (AIV) detection in the opaque biological matrices (such as avian swabs), mitigating interference issues relying on visible-range signals.

## Abstract (web summary)

Water-insensitive nanoparticles enable stable, high-contrast signals, facilitating highly sensitive avian influenza virus detection in complex biological samples with low power.

## Graphical abstract

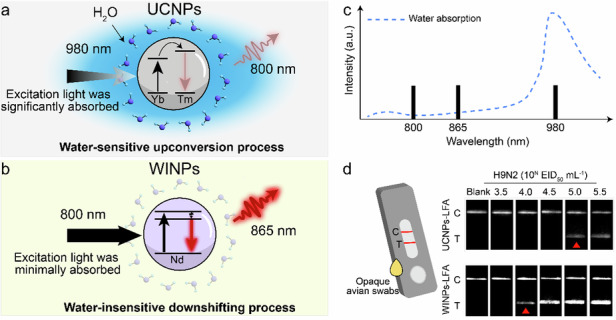

Detecting biomarkers with high sensitivity and stability in complex biological fluids remains a significant challenge in diagnostics and biomedical imaging^1,2^. Near-infrared (NIR) light offers advantages like deep tissue penetration and low autofluorescence, but the performance of lanthanide-doped upconversion nanoparticles (UCNPs), which convert NIR photons into higher-energy emissions through sequential absorption, is often hampered in aqueous environments^3,4^. UCNPs typically rely on 980 nm laser excitation, which unfortunately coincides with strong water absorption, leading to detrimental signal quenching and localized heating effects that can damage sensitive biological targets and compromise assay reliability (Fig. 1a)^5^.

**Fig. 1:**
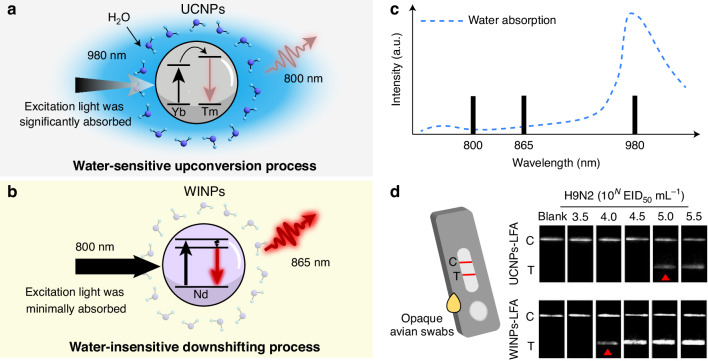
**Schematics illustrating the advantage of WINPs compared to conventional UCNPs in aqueous environments**. **a** Conventional UCNPs excited at 980 nm suffer significant water absorption leading to signal quenching. **b** WINPs excited at 800 nm show minimal water absorption, enabling stable 865 nm emission. **c** Water absorption curve, excitation and emission position of UCNPs and WINPs. **d** Application of WINPs in a low-power lateral flow assay (LFA) for sensitive detection of biomarkers like Avian Influenza Virus (AIV) in opaque avian swabs

Overcoming these limitations requires novel nanoprobe designs that are inherently resilient to water-induced quenching and can operate efficiently at low, biologically safe power density levels. Down-shifting nanoparticles, which emit lower-energy photons upon absorbing higher-energy ones, present a promising alternative pathway for the NIR biological windows^6^. Recently, Kang et al. have developed and demonstrated Water-Insensitive Down-shifting Nanoparticles (WINPs) specifically for robust performance in water-rich environments^7^. Core-shell nanoparticles (NaYF_4_:5%Nd@NaYF_4_) exhibit efficient absorption of 800 nm light and emit at 865 nm through an NIR-I-to-NIR-I down-shifting process (Fig. 1b).

Crucially, the researchers characterized the WINPs in the single-particle level. Their results confirmed their water-insensitivity: the photoluminescence lifetime profiles and the operational detection limit power density (a low 5 W cm^−2^) remained virtually identical whether the particles were measured in dry conditions or fully immersed in water. This starkly contrasts with conventional UCNPs, which showed lifetime reduction and required ~15 times higher power density to achieve a comparable signal in water due to energy transfer or absorption to water molecules. The WINPs achieved an impressive quantum yield of 22.1 ± 0.9%. Furthermore, excitation at 800 nm generated negligible thermal effects in water compared to the significant heating observed with 980 nm excitation under similar power densities (Fig. 1c).

Rapid and reliable detection of avian influenza virus (AIV) is critical for effective disease surveillance and outbreak control^8^. However, conventional lateral flow immunoassay (LFA) using colorimetric or fluorescent signals in the ultraviolet-to-visible spectrum often suffers from strong scattering and absorption in opaque and complex samples like avian swabs. Significant background interferences leading to compromised signal clarity and reduced detection reliability. To address these limitations, they integrated the WINPs into an LFA platform for the specific detection of AIV nucleocapsid protein. Remarkably, the WINP-LFA demonstrated excellent performance even under a very low-power irradiation level (100 mW cm^−2^). When tested against 65 opaque clinical samples, the assay achieved 100% sensitivity and specificity, with a detection limit about 10-fold lower than a comparable UCNP-based LFA tested under the same low-power conditions (Fig. 1d). Moreover, unlike conventional UCNPs systems that often require high excitation power leading to heat generation, WINPs provide stable, high-contrast signals without thermal drawback. This characteristic prevents heat-induced antibody denaturation and safeguards the overall reliability of the LFA.

This work addresses the challenge of water-induced luminescence quenching in NIR nanoprobes. By rationally designing an efficient NIR-I-to-NIR-I down-shifting system, the authors have created an interesting platform that functions at low power in aqueous environments. The WINP-LFA demonstrated their alternatives to conventional phosphors for developing cost-effective and sensitive biosensors. While the current NIR-I configuration demonstrates promising ex vivo sensing capabilities, its tissue penetration depth remains constrained by the fundamental optical properties of this spectral region^9^. Developing next-generation nanoprobes with excitation/emission dual NIR-II (1000–1700 nm) operation, resistance to water absorption, and high brightness is crucial for enabling non-invasive in vivo biosensing^10,11^.
